# Selection of reference genes for quantitative RT-PCR studies in striped dolphin (*Stenella coeruleoalba*) skin biopsies

**DOI:** 10.1186/1471-2199-7-32

**Published:** 2006-09-19

**Authors:** Giacomo Spinsanti, Cristina Panti, Elisa Lazzeri, Letizia Marsili, Silvia Casini, Francesco Frati, Cristina Maria Fossi

**Affiliations:** 1 Evolutionary Biology Department, University of Siena, Via A. Moro 2, 53100 Siena, Italy; 2 Environmental Sciences Department, University of Siena, Via P.A. Mattioli 4, 53100 Siena, Italy

## Abstract

**Background:**

Odontocete cetaceans occupy the top position of the marine food-web and are particularly sensitive to the bioaccumulation of lipophilic contaminants. The effects of environmental pollution on these species are highly debated and various ecotoxicological studies have addressed the impact of xenobiotic compounds on marine mammals, raising conservational concerns. Despite its sensitivity, quantitative real-time PCR (qRT-PCR) has never been used to quantify gene induction caused by exposure of cetaceans to contaminants. A limitation for the application of qRT-PCR is the need for appropriate reference genes which allow the correct quantification of gene expression. A systematic evaluation of potential reference genes in cetacean skin biopsies is presented, in order to validate future qRT-PCR studies aiming at using the expression of selected genes as non-lethal biomarkers.

**Results:**

Ten commonly used housekeeping genes (HKGs) were partially sequenced in the striped dolphin (*Stenella coeruleoalba*) and, for each gene, PCR primer pairs were specifically designed and tested in qRT-PCR assays. The expression of these potential control genes was examined in 30 striped dolphin skin biopsy samples, obtained from specimens sampled in the north-western Mediterranean Sea. The stability of selected control genes was determined using three different specific VBA applets (*geNorm*, *NormFinder *and *BestKeeper*) which produce highly comparable results. Glyceraldehyde-3P-dehydrogenase (GAPDH) and tyrosine 3-monooxygenase (YWHAZ) always rank as the two most stably expressed HKGs according to the analysis with *geNorm *and *Normfinder*, and are defined as optimal control genes by *BestKepeer*. Ribosomal protein L4 (RPL4) and S18 (RPS18) also exhibit a remarkable stability of their expression levels. On the other hand, transferrin receptor (TFRC), phosphoglycerate kinase 1 (PGK1), hypoxanthine ribosyltransferase (HPRT1) and β-2-microglobin (B2M) show variable expression among the studied samples and appear as less suitable reference genes for data normalization.

**Conclusion:**

In this work, we have provided essential background information for the selection of control genes in qRT-PCR studies of cetacean skin biopsies, as a molecular technique to investigate ecotoxicological hazard in marine mammals. Of 10 HKGs tested, those encoding for YWHAZ and GAPDH appear as the most reliable control genes for the normalization of qRT-PCR data in the analysis of striped dolphin skin biopsies. Potentially useful reference genes are also those encoding for ribosomal proteins L4 and S18.

## Background

Quantitative real-time PCR (qRT-PCR) represents a rapid and reliable method for the detection and quantification of mRNA transcription levels of a selected gene of interest (GOI). In ecotoxicological studies, qRT-PCR may be effectively used to evaluate the levels of expression of biomarker genes under the induction of xenobiotic contaminants. The sensitivity of qRT-PCR allows to work with a minimal amount of starting material, still achieving an accurate quantification of poorly transcribed mRNAs. Problems associated with the use of this assay are linked to the variability associated with the various steps of the experimental procedure, and could lead to severe misinterpretation of the results: different amounts and quality of starting material, variable enzymatic efficiencies (i.e. efficiency of retrotranscription from RNA to cDNA, and PCR efficiency) or differences between tissues or cells in overall transcriptional activity [1,2]. Among several strategies proposed [2,3], house-keeping genes (HKGs) are commonly accepted and frequently used to normalize qRT-PCR and to reduce possible errors generated in the quantification of gene expression. In this normalization strategy, internal controls are subjected to the same conditions as the RNA of interest and their expression measured by qRT-PCR. The success of this procedure is highly dependent on the choice of the appropriate control genes. Although many studies using qRT-PCR have relied upon only one endogenous control [4,5], to date the use of a single HKG appears to be insufficient, and normalization by multiple controls is required [1,6]. An ideal HKG, exposed to the same experimental protocol of the gene of interest (GOI), should present stable expression levels. If the expression of the reference gene is altered by the experimental conditions or by external factors, such as contamination, and is affected by a large variation, the noise of the assay is increased and detection of small changes becomes unfeasible, producing results that may be entirely incorrect [7]. Several works [8-10] prove how some of the most commonly used HKGs can not always be considered as reliable controls and/or they show different behaviour in various tissues [11], emphasizing the importance of preliminary evaluation studies, aimed at identifying the most stable HKGs in different organisms. The number of articles concerning the evaluation and selection of the best HKGs for each single experiment [11-19] is rapidly increasing, together with the number of softwares which use statistical methods to evaluate stability of selected HKGs [1,6,20]. Some HKGs (those encoding for Act-B, GAPDH, HPRT1 and 18S ribosomal RNA) have been used as reference for many years in Northern blots, RNase protection tests and conventional quantitative PCR (qPCR) [3]. With the introduction of qRT-PCR, other ordinarily used HKGs, involved with basic and ubiquitous cellular functions and belonging to different functional classes, have been introduced [21-25].

Top predators, such as odontocete cetaceans, are known to accumulate high concentrations of persistent organic pollutants (POPs), including endocrine disrupting chemicals (EDCs), thereby incurring in high toxicological hazard [26]. EDCs mime sex steroid hormones, both androgens and estrogens, binding to hormone receptors and influencing cellular pathways [27]. Xenobiotic compounds exhibit lipophilic properties and tend to accumulate preferentially in the fat tissue of top predators. Striped dolphins (*Stenella coeruleoalba*) inhabiting the Mediterranean basin appear to be particularly exposed to the bioaccumulation of OCs if compared to other worldwide distributed cetacean populations [27]. In addition, a geographical contamination trend was accurately described in the Mediterranean basin for the striped dolphin [28], where levels of OCs in the body of these animals decrease from north-west to south-east. It is evident that cetaceans inhabiting the Ligurian Sea appear to be much more exposed to OCs if compared to those sampled in the Eolian Archipelago and the Ionian Sea [27]. Specimens of the striped dolphin from the Ligurian Sea have been chosen as a test population to validate qRT-PCR studies aimed at a better understanding of the role of POPs in the induction of potential biomarker genes.

Previous ecotoxicological studies have assessed that cetacean skin biopsies constitute a suitable, non-lethal, biological material for the assessment of toxicological hazard in Mediterranean marine mammals [29,30]. They enable the evaluation of levels of OCs with endocrine disrupting capacity and of Cytochrome P450 (CYP1A1) induction, as an early warning sign of exposure to organochlorines [26-32].

Despite its sensitivity and reliability, qRT-PCR has never been used in cetacean skin biopsies to investigate gene induction. In this context, our work constitutes the first effort towards the routine application of this molecular technique for the evaluation of toxicological hazard in cetacean species. Ten among the most common HKGs have been described and ranked, according to the stability of their expression, in skin biopsies of the striped dolphin. The animals were sampled in the "Pelagos Sanctuary for Mediterranean Marine Mammals", a special marine protected area extending for about 90.000 km^2 ^in the north-western Mediterranean Sea between Italy, France and the Sardinia Island, encompassing Corsica Island and the Tuscan Archipelago.

## Results and discussion

We obtained the partial sequence of ten of the most commonly used housekeeping genes: β-Actin (Act-B), glyceraldehyde-3P-dehydrogenase (GAPDH), hypoxanthine ribosyltransferase (HPRT1), β-2-microglobin (B2M), phosphoglycerate kinase 1 (PGK1), succinate dehydrogenase complex, subunit A (SDHA), transferrin receptor (TFRC), tyrosine 3-monooxygenase (YWHAZ), L4 (RPL4) and S18 (RPS18) ribosomal proteins. Sequence information, deposited in GenBank under the Accession Numbers shown in Table 1, was used to design RT-PCR primers (Table 2). Target amplicons, never exceeding 171 bp, were sequenced to confirm specificity of the products. The efficiency of real-time amplifications for each single primer pair was determined in RT-PCR using 1:5 cDNA serial dilutions retrotranscribed from 1 μg total RNA isolated in striped dolphin skin biopsies (Table 2). Expression patterns of the ten HKGs have been subsequently evaluated in skin biopsies (*n *= 30) obtained using non-lethal techniques, in a Mediterranean sub-population of the striped dolphin from the Ligurian Sea.

### RNA quality

All RNA samples were examined as to their concentration, purity and integrity. Based on absorbance ratio at 260/280 nm and at 230/260 nm, all samples were pure, free from protein and organic pollutants derived from RNA extraction. Overall sample integrity was confirmed by denaturing formaldehyde agarose gel electrophoresis, showing sharp and intense 18S and 28S ribosomal RNA bands with a total absence of smears.

### Expression levels of candidate reference genes

**Figure 1:**
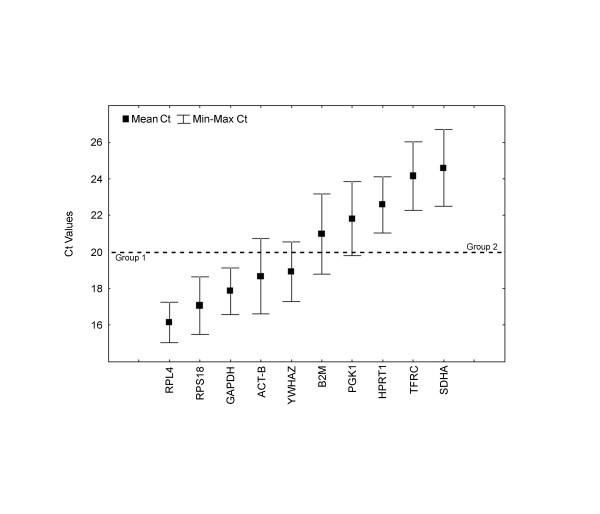
**Expression levels of candidate HKGs**. Expression levels of candidate control genes in the tested striped dolphin skin biopsies (*n *= 30). Values are given as qRT-PCR cycle threshold numbers (Ct values). Boxes represent mean Ct values and whiskers the range of Ct values in the 30 samples. The arbitrary line at Ct 20 distinguishes two groups of similarly expressed housekeeping genes.

The 10 studied HKGs displayed a relatively wide range of expression level, from the lowest mean Ct values (16.1) in RPL4, to the highest (24.6) in SDHA. We grouped the expression levels in two arbitrary categories as shown in Figure 1. Highly expressed genes have mean Ct values below 20 cycles (RPL4, RPS18, GAPDH, Act-B and YWHAZ), and lowly expressed genes have mean Ct values above 20 cycles (PGK1, B2M, HPRT1, TFRC and SDHA). Individual HKGs have different expression levels across all studied samples, as shown in Figure 1. RPL4 and GAPDH have the smallest gene expression variation (below 3 cycles) while, PGK1, Act-B, SDHA and B2M are the genes with the most variable levels of expression (over 4 cycles).

### Data analysis

The data obtained for each skin biopsy and each HKG were analysed using three different VBA applets, *geNorm *[1,33], *NormFinder *[20,34] and *BestKeeper *[6,35].

**Figure 2:**
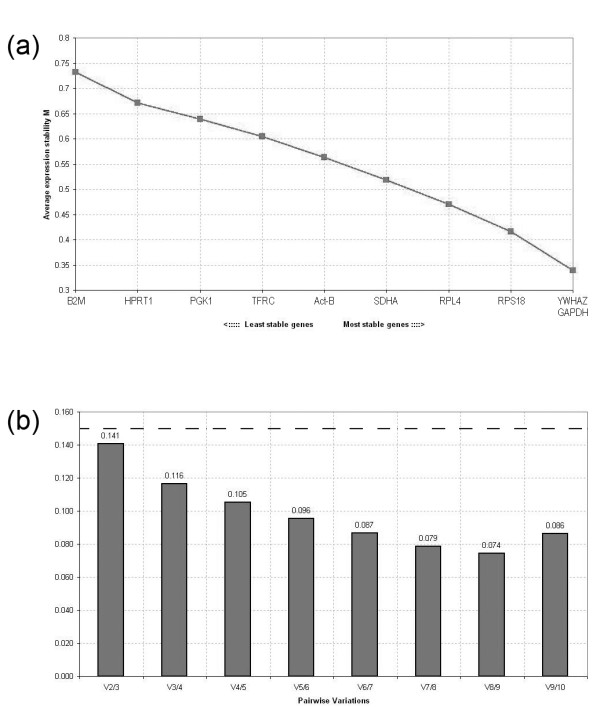
***geNorm *output charts**. (a) average expression stability measure (M) of control genes during stepwise exclusion of the least stable control genes; (b) determination of the optimal number of control genes for normalization calculated on the basis of the pair-wise variation (V) analysis; V values under 0.15 threshold line indicate no need to include further HKG for calculation of a reliable normalization factor.

*geNorm *provides a ranking of the tested genes, based on their expression stability, determining the two most stable HKGs or a combination of multiple stable genes for normalization. Selected HKGs were ranked according to the determined control gene-stability measure (M, average pair-wise variation of a particular gene with all other control genes), from the most stable (lowest M values) to the least stable (highest M values): GAPDH/YWHAZ – RPS18 – RPL4 – SDHA – Act-B – TFRC – PGK1 – HPRT1 – B2M (Table 3; Figure 2a). All studied genes reach a high expression stability with low M values, less than 1, below the default limit of M = 1.5 [1]. Interestingly, the expression of the gene encoding for Act-B, widely used as a HKG in many studies, appears to be less stable than other genes in skin samples where Act-B should be constitutively expressed at high levels, presenting a large expression variation as calculated by Ct values. It is also remarkable that the two ribosomal proteins (S18 and L4) exhibit a similar behaviour (ranking as the 3^rd ^and 4^th ^most stably expressed genes, respectively) and are characterized by highly comparable average M values (Table 3). Additionally, the assessment of the normalization factor allows the identification of the optimal number of control genes. The *geNorm *software suggests that an accurate normalization factor of qRT-PCR data can be calculated by using these two most stably expressed genes (YWHAZ and GAPDH). As shown in Figure 2b, the addition of further HKGs will not significantly affect the reliability of the determined normalization factor, yielding a V_2/3 _value (pair-wise variation between two sequential normalization factors) of 0.141, lower than the default cut-off value of 0.15 [1]. According to the *geNorm *stability rank of the HKGs studied, the third gene to include in the calculation of a reliable normalization factor should be chosen between the two ribosomal protein genes S18 and L4 (Table 3; Figure 2a).

*NormFinder *is another VBA applet based on an algorithm for identifying the optimal normalization gene(s) among a set of candidates. It ranks the set of candidate genes according to their expression stability value in a given sample set and a given experimental design [20]. The results of the *NormFinder *analysis applied to our data are shown in Table 4. This ranking appears to be identical to the one previously determined using *geNorm*, except for the position of the two ribosomal protein genes S18 and L4, which are inverted. YWHAZ, GAPDH, RPL4 and RPS18 still occupy the highest positions, while TFRC, PGK1, HPRT1 and B2M are equally defined as the least reliable controls. As one would expect given their identical cellular function, the genes encoding for the ribosomal proteins S18 and L4 occupy the same position as in the *geNorm *rank.

*BestKeeper *is an Excel-based tool determining the "optimal" HKGs by using a pair-wise correlation analysis of all pairs of candidate genes, and calculating the geometric mean of the "best" suited ones [6]. The first analysis of the data, based on the inspection of raw Ct values calculated variations for all the HKGs in the 30 biopsies samples (SD [± C_t_] and CV [%C_t_]), shows an overall stability in gene expression (Table 5). Since all tested HKGs exhibit a SD value lower than 1, none of them can be clearly considered inconsistent, and therefore excluded from the calculation of the *BestKeeper *index [6]. For this reason, all control genes have been retained in the calculation of the *BestKeeper *index, which finally exhibit a moderate SD variation of 0.51. Most important, *BestKeeper *allows a comparative analysis across HKGs, by estimating correlations in the expression levels between all the possible candidates. Highly correlated control genes are combined into an index. Afterwards, the pair-wise correlation between genes and the correlation between each gene and the index are calculated, describing the relation between the index and the contributing HKG [6]. The 10 control genes tested in our analysis correlate well one with another, and also if compared with the *BestKeeper *index (Table 6). The best correlation between the HKGs and the *BestKeeper *index is obtained for YWHAZ (r = 0.938), followed by Act-B and GAPDH (Table 6). The statistically significant correlation shown by Act-B (r = 0.935) with the *BestKeeper *index (Table 6) appears inconsistent with the performance of this gene as assessed by *geNorm *and *NormFinder*. YWHAZ and GAPDH are still ranked as two of the most reliable control genes, showing respectively the best (0.938) and the third best (0.927) correlation values (Table 6). It is also remarkable how TFRC, PGK1, HPRT1 and B2M are again classified as the least reliable HKGs, showing the worst correlations with the determined *BestKeeper *index (Table 6). As previously mentioned, the genes encoding for the ribosomal proteins L4 and S18, which share identical cellular functions, appear to be strictly correlated and show highly comparable SD and r values (Table 5 and 6).

Calculation of the InVar parameter (as described in [6]) shows an overall good sample integrity.

## Conclusion

This work constitutes the first effort for the selection of optimal control genes in qRT-PCR studies designed for the assessment of ecotoxicological hazard in cetacean populations. The three softwares tested (*geNorm*, *NormFinder *and *BestKeeper*), based on different algorithms and analytical procedures, produced highly comparable results.

Our study let us conclude that GAPDH and YWHAZ are the most reliable HKGs of this set and we therefore strongly recommend their use in future qRT-PCR studies on the striped dolphin. However, it is common opinion that the reliability of the normalization factor increases together with the number of stably expressed HKGs included in its calculation and the use of at least three HKGs for the correct normalization of qRT-PCR data has been proposed [1]. Our analysis suggests that the ribosomal protein genes RPL4 and RPS18 constitute additional useful HKGs.

On the other hand, TFRC, PGK1, HPRT1 and B2M show unstable expression patterns and are always classified as the least reliable control genes of this group.

It appears, from Figure 1, that the best reference genes selected from this study are also those with higher levels of expression (lower Ct values). While highly expressed genes might in principle exhibit lower levels of inter-individual variation, it should be pointed out that the softwares tested here do not assess stability as a function of variation in Ct values. In fact, the reliability of a given HKG is measured as a function of the variation of the ratios between the level of expression of pairs of HKGs across all samples. It is, therefore, a relative measure (to all other HKGs tested), not an absolute measure of variation of expression of a single control gene across samples. The gene encoding for Act-B is indeed one of the most commonly used HKGs, and it has found application in expression studies of various animal taxa. In our study, however, this gene is outperformed by several other genes (mostly GAPDH, YWHAZ, RPL4 and RPS18) as an optimal HKG. It is worth noting that Act-B shows a significant correlation (Table 6) with the *BestKeeper *index, comparable to that of GAPDH and YWHAZ, an outcome that would suggest some potential usefulness for this gene.

Ideally, in an experimental application, one could choose to use preferentially HKGs that have the same expression levels as the target gene, in order to enhance the uniformity of the analysis. However, two considerations must be made in this context. First, the use of target genes with extremely low physiological levels of expression should always be avoided, because of the considerable variance due to experimental fluctuations. Second, by definition, the expression levels of a good target gene are supposed to be under a strong influence by any contaminant whose effects are being studied. It is therefore likely that these levels vary across a wide range of Ct values. Nevertheless, if a target gene with very low level of expression is chosen, we may recommend the inclusion, among the control genes, of SDHA, which has demonstrated to possess a sub-optimal suitability as a reference gene.

## Methods

### Skin biopsies sampling

Sub-samples of skin biopsies (around 30 mg in weight) of 30 different specimens of the striped dolphin were obtained during several sampling trips in the Ligurian Sea, in the summer of 2005 (International CITES permit: IT 007). Dolphins were sampled from the prow of the boat using biopsy tips mounted on an aluminium pole [29]. Biopsy samples were immediately stored in RNA later (Ambion) at 4°C overnight and, subsequently, at -80°C.

### Total RNA and genomic DNA isolation and characterization

Skin biopsies were homogenized with a TissueLyser (Qiagen, Hilden, Germany).

Total RNA was subsequently extracted using the RNeasy Fibrous Tissue Mini Kit (Qiagen), according to the manufacturer's instructions. Genomic DNA was eliminated by a DNase-on-column treatment supplied with the kit. The quantity of extracted RNA was assessed with the NanoDrop^® ^ND-1000 UV-Vis Spectrophotometer (NanoDrop Technologies, Wilmington, DE, USA); absorbance ratio at 260/280 nm and 260/230 nm was used to assess purity of the RNA samples [36]. The integrity of the samples was determined by denaturing agarose gel (1.2%) electrophoresis and ethidium bromide staining.

Genomic DNA was isolated from homogenized skin biopsies using the Wizard^® ^SV Genomic DNA Purification System (Promega, Madison, Wisconsin, USA). Final elution was in 80 μl Nuclease Free Water (Promega) and 1 μl was subsequently used in PCR amplification reactions.

### Sequencing HKGs

Ten potential HKGs have been evaluated in this study: β-Actin (Act-B), glyceraldehyde-3P-dehydrogenase (GAPDH), hypoxanthine ribosyltransferase (HPRT1), β-2-microglobin (B2M), phosphoglycerate kinase 1 (PGK1), succinate dehydrogenase complex, subunit A (SDHA), transferrin receptor (TFRC), tyrosine 3-monooxygenase (YWHAZ), and the ribosomal proteins L4 (RPL4) and S18 (RPS18). To reduce possible errors due to co-regulation of different genes, we decided to include in our analysis HKGs involved in different cellular functions (Table 1). The only potential co-regulated genes are those encoding for the two ribosomal proteins S18 and L4. Partial nucleotide sequences of all selected HKGs have been determined in the striped dolphin. PCR primers were designed in conserved regions by comparing sequences of all genes available from GenBank for several mammal species. Alignments were performed with ClustalW [37,38] and BioEdit [39]. Both alignments and primer sequences are available from the authors upon request. Primers were used in PCR reactions on cDNA retrotranscribed from striped dolphin skin biopsies. Retrotranscriptions were carried out with 1 μg total RNA using iScript cDNA synthesis kit (Bio-Rad, Hercules, California, USA), according to the manufacturer's instructions. 1 μl cDNA was used as template in a standard 25 μl PCR reaction, using 10 μM of each primer and 0.05 U *Taq *polimerase (Promega). In order to extend the sequence information for some genes, 3'-RACE experiments were carried out for Act-B, RPS18 and GAPDH genes, using the 3'-RACE System for Rapid Amplification of cDNA Ends (Invitrogen, Carlsbad, California, USA). A 5'-RACE experiment was also performed with the 5'-RACE System (Invitrogen) on the cDNA of the B2M gene.

Amplification products were first loaded and checked on a 1% agarose gel, and sequenced on a automatic sequencer CEQ8000XL (Beckman Coulter, Fullerton, California, USA) using DTCS system, purified with Wizard SV Gel and PCR Clean-Up System (Promega). Electropherograms and sequences were compared and manually corrected using Sequencher 4.5 (GeneCodes, Ann Arbor, Michigan, USA). Sequences, checked for their specificity using Blast [40], were deposited in GenBank (see Table 1 for Accession Numbers).

### Design of RT-PCR primers

PCR primers for real-time assays were designed on the determined nucleotide sequences of each HKG using Beacon Designer 2.06 (Premier Biosoft International, Palo Alto, California, USA). Special attention was given to primer length, annealing temperature, base composition and 3'-end stability. To ensure optimal DNA polymerization efficiency, amplicons length ranged between 84 and 171 bp. Exon and intron boundaries were determined aligning sequenced genes with corresponding human genomic sequences downloaded from GenBank. Position of introns in the HKGs, deduced from the alignments with *Homo sapiens*, was subsequently confirmed by PCR on striped dolphin genomic DNA. As expected [41,42], intron position was highly conserved between cetaceans and human. On the basis of this knowledge, except for GAPDH, we designed RT-PCR primers located on different exons or directly spanning exon-exon junction of each cDNA (Table 2). After preliminary real-time assays, the optimal primer concentration appeared to be 300 mM, which generates the lowest Ct value and a sharp peak, with no amplification of non-specific products or primer-dimer artifacts (see additional files 1, 2, 3, 4, 5, 6, 7, 8, 9 and 10). For each different pair of primers, efficiency of RT-PCR (E), slope values, and correlation coefficients (R^2^) were determined, using serial 1:5 dilutions of template cDNA, on a iQ5 machine (Bio-Rad) (Table 2). Products were subsequently run on 2% agarose gel to check for size specificity, and, eventually, sequenced.

### Real-time quantitative PCR (qRT-PCR)

To avoid any genomic DNA contamination during qRT-PCR, retrotranscription of 1 μg total RNA was performed with the QuantiTect Reverse Transcription Kit (Qiagen), according to the manufacturer's instructions. This kit ensures complete digestion of genomic DNA by a brief incubation of the sample at 42°C with a specific Wipeout buffer before retrotranscription. For each of the thirty samples, 1 μl of treated RNA was collected before proceeding with the retrotranscription step, and stored at -80°C. Treated RNA samples have been subsequently used in RT-PCR, as internal controls to check for potential genomic DNA contamination.

The second part of this two-step RT-PCR protocol was characterized by real-time amplifications, using SYBR Green detection chemistry, run in triplicate on 96-wells reaction plates with the iQ5 machine (Bio-Rad). Reactions were prepared in a total volume of 20 μl containing: 1 μl cDNA, 0.8 μl of each 10 μM primer (300 mM each; Invitrogen), 10 μl of iQ™ SYBR^® ^Green Supermix (Bio-Rad) and 8 μl RNase,/DNase-free sterile water (Qiagen). Blank controls were run in triplicate for each master mix. The cycle conditions were set as follows: initial template denaturation at 95°C for 1 min, followed by 40 cycles of denaturation at 95°C for 10 s, and combined primer annealing/elongation at 60°C for 30 s, as described in [4]. This cycle was followed by a melting curve analysis, ranging from 56°C to 95°C, with temperature increasing steps of 0.5°C every 10 s.

Baseline and threshold values were automatically determined for all plates using the Bio-Rad iQ5 Software 1.0. In order to ensure comparability between data obtained from different experimental plates, the threshold value has been subsequently manually set to the value corresponding to the arithmetic mean between the automatically determined thresholds annotated previously; then all data have been reanalyzed. Raw Ct values were transformed to quantities using an Excel spreadsheet generated by the authors, based on the comparative Ct method. The data obtained have been converted into correct input files, according to the requirements of the software, and analysed using *geNorm *(version 3.4), *NormFinder *(version 0.953) and *BestKeeper *(version 1)VBA applets.

## Authors' contributions

GS performed all the experimental procedure and was primary author of the manuscript. CP and EL participated in the experimental process and data analysis. LM and MCF provided the samples. MCF, FF, LM and SC supervised the study design and contributed to writing the manuscript.

## Supplementary Material

Additional File 1 **Melting curve analyses obtained for the Act-B gene**. Melting curve analyses image (jpg format) collected using the iQ5 Optical System Software 1.0 (Bio-Rad) during calibration experiments of the selected primer pair for the Act-B gene. Data were obtained using 1:5 dilutions of template cDNA (retrotranscribed from striped dolphin skin biopsy isolated total RNA) on a iQ5 machine (Bio-Rad). Click here for file

Additional File 2 **Melting curve analyses obtained for the GAPDH gene**. Melting curve analyses image (jpg format) collected using the iQ5 Optical System Software 1.0 (Bio-Rad) during calibration experiments of the selected primer pair for the GAPDH gene. Data were obtained using 1:5 dilutions of template cDNA (retrotranscribed from striped dolphin skin biopsy isolated total RNA) on a iQ5 machine (Bio-Rad). Click here for file

Additional File 3 **Melting curve analyses obtained for the HPRT1 gene**. Melting curve analyses image (jpg format) collected using the iQ5 Optical System Software 1.0 (Bio-Rad) during calibration experiments of the selected primer pair for the HPRT1 gene. Data were obtained using 1:5 dilutions of template cDNA (retrotranscribed from striped dolphin skin biopsy isolated total RNA) on a iQ5 machine (Bio-Rad). Click here for file

Additional File 4 **Melting curve analyses obtained for the B2M gene**. Melting curve analyses image (jpg format) collected using the iQ5 Optical System Software 1.0 (Bio-Rad) during calibration experiments of the selected primer pair for the B2M gene. Data were obtained using 1:5 dilutions of template cDNA (retrotranscribed from striped dolphin skin biopsy isolated total RNA) on a iQ5 machine (Bio-Rad). Click here for file

Additional File 5 **Melting curve analyses obtained for the PGK1 gene**. Melting curve analyses image (jpg format) collected using the iQ5 Optical System Software 1.0 (Bio-Rad) during calibration experiments of the selected primer pair for the PGK1 gene. Data were obtained using 1:5 dilutions of template cDNA (retrotranscribed from striped dolphin skin biopsy isolated total RNA) on a iQ5 machine (Bio-Rad). Click here for file

Additional File 6 **Melting curve analyses obtained for the SDHA gene**. Melting curve analyses image (jpg format) collected using the iQ5 Optical System Software 1.0 (Bio-Rad) during calibration experiments of the selected primer pair for the SDHA gene. Data were obtained using 1:5 dilutions of template cDNA (retrotranscribed from striped dolphin skin biopsy isolated total RNA) on a iQ5 machine (Bio-Rad). Click here for file

Additional File 7 **Melting curve analyses obtained for the TFRC gene**. Melting curve analyses image (jpg format) collected using the iQ5 Optical System Software 1.0 (Bio-Rad) during calibration experiments of the selected primer pair for the TFRC gene. Data were obtained using 1:5 dilutions of template cDNA (retrotranscribed from striped dolphin skin biopsy isolated total RNA) on a iQ5 machine (Bio-Rad). Click here for file

Additional File 8 **Melting curve analyses obtained for the YWHAZ gene**. Melting curve analyses image (jpg format) collected using the iQ5 Optical System Software 1.0 (Bio-Rad) during calibration experiments of the selected primer pair for the YWHAZ gene. Data were obtained using 1:5 dilutions of template cDNA (retrotranscribed from striped dolphin skin biopsy isolated total RNA) on a iQ5 machine (Bio-Rad). Click here for file

Additional File 9 **Melting curve analyses obtained for the RPL4 gene**. Melting curve analyses image (jpg format) collected using the iQ5 Optical System Software 1.0 (Bio-Rad) during calibration experiments of the selected primer pair for the RPL4 gene. Data were obtained using 1:5 dilutions of template cDNA (retrotranscribed from striped dolphin skin biopsy isolated total RNA) on a iQ5 machine (Bio-Rad). Click here for file

Additional File 10 **Melting curve analyses obtained for the RPS18 gene**. Melting curve analyses image (jpg format) collected using the iQ5 Optical System Software 1.0 (Bio-Rad) during calibration experiments of the selected primer pair for the RPS18 gene. Data were obtained using 1:5 dilutions of template cDNA (retrotranscribed from striped dolphin skin biopsy isolated total RNA) on a iQ5 machine (Bio-Rad). Click here for file

## Figures and Tables

**Table 1:** GenBank Accession Numbers of partially sequenced genes in *Stenella coeruleoalba*.

Gene Symbol	Gene Name	Function	Gene Aliases	Acc. Numb.
Act-B	β-actin	Cytoskeletal structural protein		DQ404535
B2M	β-2-microglobin	Cytoskeletal protein involved in cell locomotion		DQ533609
GAPDH	glyceraldehyde-3-phosphate dehydrogenase	Glycolitic enzyme	G3PD; GAPDH	DQ404538
HPRT1	hypoxantine phosphoribosyltransferase 1	Metabolic salvage of purines in mammals	HPRT1; HGPRT	DQ533610
PGK1	phosphoglycerate kinase 1		PGKA	DQ533611
RPL4	ribosomal Protein L4	Member of ribosome proteins		DQ404536
RPS18	ribosomal Protein S18	Member of ribosome proteins		DQ404537
SDHA	succinate dehydrogenase complex	Electron transporter in the TCA cycle and respiratory chain	FP, SDH2, SDHF	DQ533607
TFRC	transferrin receptor	Transferrin receptor	TFR; p90; CD71; TFR1; Trfr; Mtvr1; Mtvr-1	DQ533608
YWHAZ	tyrosine 3-monooxygenase	Signal transduction by binding to phosphorilated serine residues on a variety of signaling molecules	KCIP-1	DQ404539

**Table 2:** Details of primers and amplicons for each of the 10 evaluated genes.

Gene	Forward Primer Sequence [5'→3']	Position in cDNA	Reverse Primer Sequence [5'→3']	Position in cDNA	Amplicon Length	E%	R^2^
Act-B	CCATCGGCAATGAGCGGTTCC	3^rd ^Exon	CGTGTTGGCATACAGGTCCTTCC	4^th ^Exon	146bp	97.9	0.997
B2M	TGGCTCCTTTCGTGACCTTGG	1^st ^Exon	GTCGTGAGTAAACCTGAACCTTCG	2^nd ^Exon	96bp	99	0.996
GAPDH	CAAGGCTGTGGGCAAGGTCATC	7^th ^Exon	TTCTCCAGGCGGCAGGTCAG	7^th ^Exon	111bp	97.4	0.995
HPRT1	GTCAAGCAGCATAATCCAAAGATG	6^th ^Exon	GTCTGGTCTATATCCAACACTTCG	7^th ^Exon	84bp	98.1	0.998
PGK1	ACAATGGAGCCAAGTCAG	3^rd ^Exon	CACGCAGTCCTTCAAGAAC	4^th ^Exon	146bp	97.4	0.996
RPL4	CAGACCTTAGCAGAATCTTGAAAAGC	8^th ^Exon	CCTGGCGAAGAATGGTGTTCC	9^th ^Exon	171bp	99.9	0.998
RPS18	CAATTAAGGGTGTGGGGCGAAG	2^nd^/3^rd ^Exon	TCTTGTATTGGCGTGGATTCTGC	4^th ^Exon	141bp	100.8	0.999
SDHA	TCTTTCCCACCAGGTCACACAC	3^rd ^Exon	CCAGTCGGAGCCCTTCACG	4^th ^Exon	119bp	88.2	0.993
TFRC	ACCATCTCAGTCATCAGGATTG	8^th ^Exon	CATTCTTGTTCTGTGAGGATACC	9^th ^Exon	152bp	98.1	0.998
YWHAZ	AAATGAAAGGAGACTACTACCGCTA	2^nd ^Exon	AGACCCAATCTGATAGGATGTGTTG	3^rd ^Exon	151bp	95.9	0.994

Primers are located in different exons, except for GAPDH, where primers are located in the same exon. E is a measure of real-time reaction efficiency and R^2 ^indicates reproducibility of the reaction.

**Table 3:** Candidate reference genes for normalization of qRT-PCR ranked according to their expression stability (calculated as the average M value after stepwise exclusion of worst scoring genes) by the geNorm VBA applet.

Ranking Order	Gene Name	Average M value
1/2	GAPDH/YWHAZ	0.338
3	RPS18	0.416
4	RPL4	0.469
5	SDHA	0.517
6	Act-B	0.563
7	TFRC	0.604
8	PGK1	0.638
9	HPRT1	0.671
10	B2M	0.732

**Table 4:** Candidate reference genes for normalization of qRT-PCR listed according to their expression stability calculated by the *NormFinder *VBA applet.

Ranking Order	Gene Name	Stability Value
1	GAPDH	0.155
2	YWHAZ	0.157
3	RPL4	0.278
4	RPS18	0.322
5	SDHA	0.343
6	Act-B	0.368
7	TFRC	0.385
8	PGK1	0.420
9	HPRT1	0.466
10	B2M	0.588

**Table 5:** Results from *BestKeeper *descriptive statistical analysis.

	Act-B	B2M	YWHAZ	TFRC	SDHA	RPS18	RPL4	PGK1	HPRT1	GAPDH
N	30	30	30	30	30	30	30	30	30	30
GM [Ct]	18.66	21.23	18.95	24.10	24.82	16.83	16.13	21.36	23.04	17.82
AM [Ct]	18.69	21.26	18.97	24.11	24.85	16.84	16.14	21.37	23.05	17.83
Min [Ct]	16.60	18.79	17.29	22.26	22.49	15.49	15.05	19.78	21.05	16.59
Max [Ct]	20.72	23.17	20.56	26.04	26.69	18.63	17.25	23.83	24.12	19.12
SD [± Ct]	0.85	0.84	0.58	0.67	0.90	0.53	0.39	0.67	0.38	0.55
CV [% Ct]	4.57	3.97	3.07	2.77	3.60	3.13	2.43	3.13	1.67	3.10

Abbreviations: N: number of samples; GM [Ct]: geometric mean of Ct; AM [Ct] arithmetic mean of Ct; Min [Ct] and Max [Ct]: extreme values of Ct; SD [± Ct]: standard deviation of the Ct; CV [% Ct]: coefficient of variance expressed as a percentage on the Ct level.

**Table 6:** Results from *BestKeeper *correlation analysis.

BestKeeper vs.	Act-B	B2M	YWHAZ	TFRC	SDHA	RPS18	RPL4	PGK1	HPRT1	GAPDH
coeff. of corr. [r]	0.935*	0.668	0.938*	0.770	0.884	0.802	0.783	0.737	0.447	0.927*
p-value	0.001	0.001	0.001	0.001	0.001	0.001	0.001	0.001	0.013	0.001

Measures of the correlation coefficients between each control gene and the *BestKeeper *index. HKGs strictly correlated between them and the index are marked by *.
