# Supplementary figures and images for: Selection of reference genes for quantitative RT-PCR studies in striped dolphin (Stenella coeruleoalba) skin biopsies
Source: BMC Mol Biol. 2006 Sep 19;7:32. doi: 10.1186/1471-2199-7-32 (PMC1599742; doi:10.1186/1471-2199-7-32)

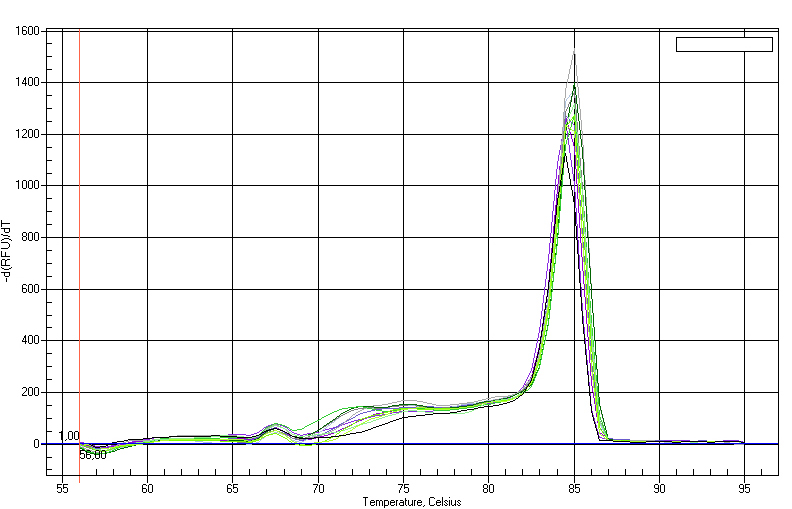

Supplement: Additional File 1 — Melting curve analyses obtained for the Act-B gene. Melting curve analyses image (jpg format) collected using the iQ5 Optical System Software 1.0 (Bio-Rad) during calibration experiments of the selected primer pair for the Act-B gene. Data were obtained using 1:5 dilutions of template cDNA (retrotranscribed from striped dolphin skin biopsy isolated total RNA) on a iQ5 machine (Bio-Rad). [file 1471-2199-7-32-S1.jpeg]

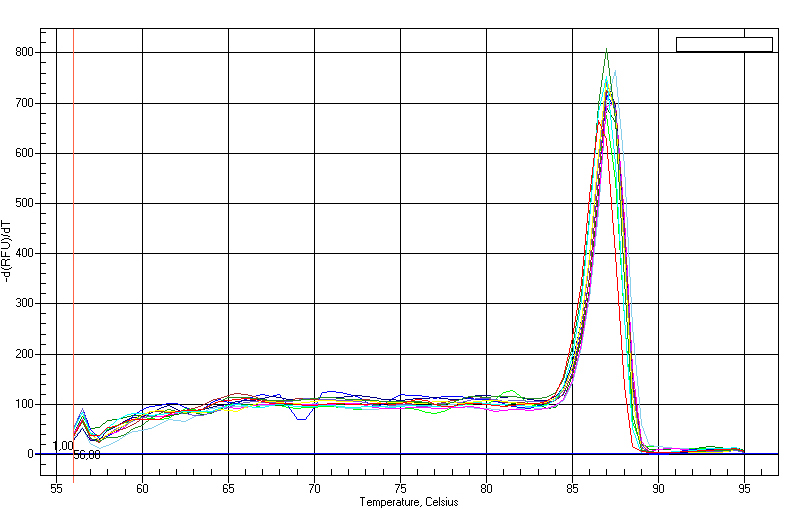

Supplement: Additional File 2 — Melting curve analyses obtained for the GAPDH gene. Melting curve analyses image (jpg format) collected using the iQ5 Optical System Software 1.0 (Bio-Rad) during calibration experiments of the selected primer pair for the GAPDH gene. Data were obtained using 1:5 dilutions of template cDNA (retrotranscribed from striped dolphin skin biopsy isolated total RNA) on a iQ5 machine (Bio-Rad). [file 1471-2199-7-32-S2.jpeg]

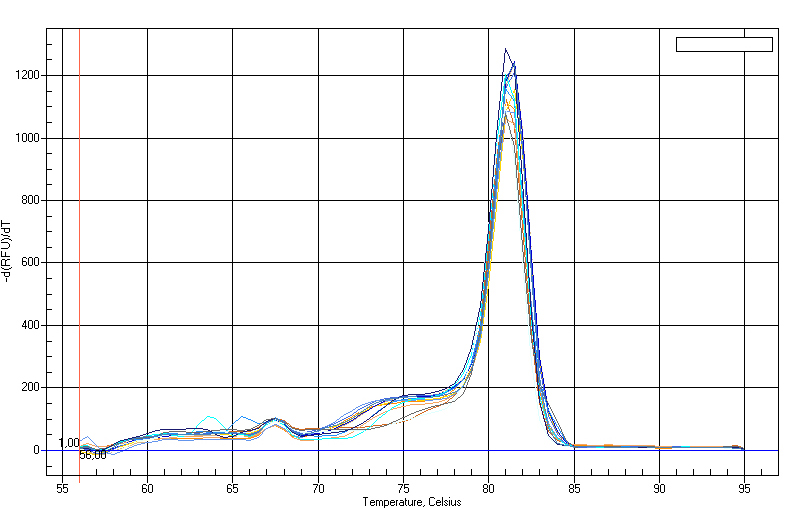

Supplement: Additional File 3 — Melting curve analyses obtained for the HPRT1 gene. Melting curve analyses image (jpg format) collected using the iQ5 Optical System Software 1.0 (Bio-Rad) during calibration experiments of the selected primer pair for the HPRT1 gene. Data were obtained using 1:5 dilutions of template cDNA (retrotranscribed from striped dolphin skin biopsy isolated total RNA) on a iQ5 machine (Bio-Rad). [file 1471-2199-7-32-S3.jpeg]

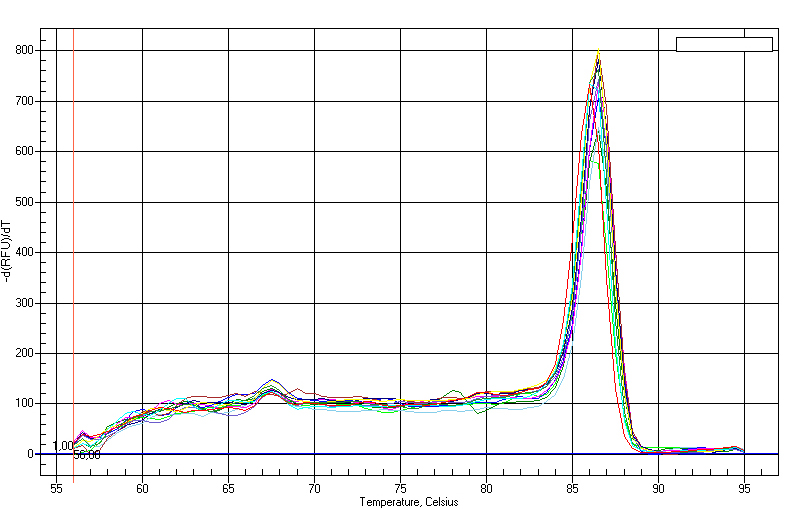

Supplement: Additional File 4 — Melting curve analyses obtained for the B2M gene. Melting curve analyses image (jpg format) collected using the iQ5 Optical System Software 1.0 (Bio-Rad) during calibration experiments of the selected primer pair for the B2M gene. Data were obtained using 1:5 dilutions of template cDNA (retrotranscribed from striped dolphin skin biopsy isolated total RNA) on a iQ5 machine (Bio-Rad). [file 1471-2199-7-32-S4.jpeg]

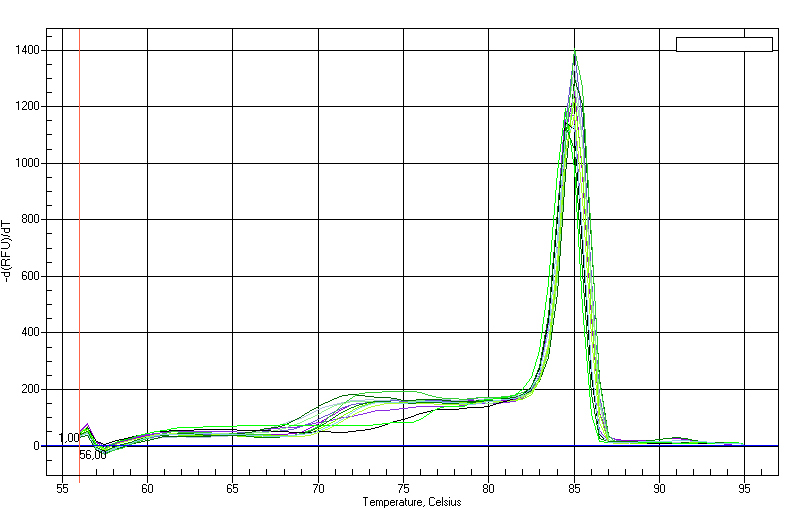

Supplement: Additional File 5 — Melting curve analyses obtained for the PGK1 gene. Melting curve analyses image (jpg format) collected using the iQ5 Optical System Software 1.0 (Bio-Rad) during calibration experiments of the selected primer pair for the PGK1 gene. Data were obtained using 1:5 dilutions of template cDNA (retrotranscribed from striped dolphin skin biopsy isolated total RNA) on a iQ5 machine (Bio-Rad). [file 1471-2199-7-32-S5.jpeg]

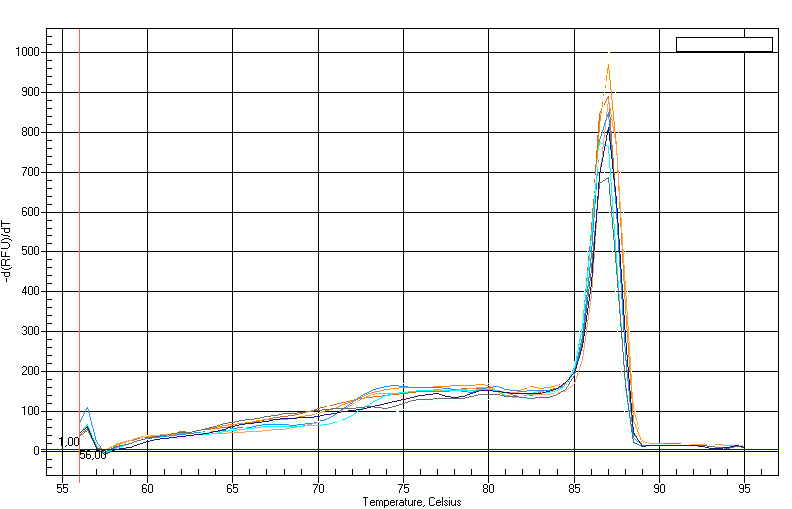

Supplement: Additional File 6 — Melting curve analyses obtained for the SDHA gene. Melting curve analyses image (jpg format) collected using the iQ5 Optical System Software 1.0 (Bio-Rad) during calibration experiments of the selected primer pair for the SDHA gene. Data were obtained using 1:5 dilutions of template cDNA (retrotranscribed from striped dolphin skin biopsy isolated total RNA) on a iQ5 machine (Bio-Rad). [file 1471-2199-7-32-S6.jpeg]

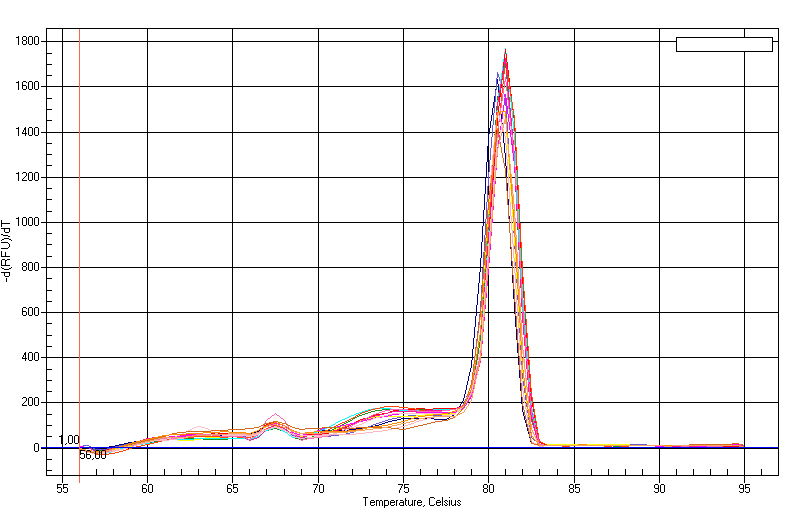

Supplement: Additional File 7 — Melting curve analyses obtained for the TFRC gene. Melting curve analyses image (jpg format) collected using the iQ5 Optical System Software 1.0 (Bio-Rad) during calibration experiments of the selected primer pair for the TFRC gene. Data were obtained using 1:5 dilutions of template cDNA (retrotranscribed from striped dolphin skin biopsy isolated total RNA) on a iQ5 machine (Bio-Rad). [file 1471-2199-7-32-S7.jpeg]

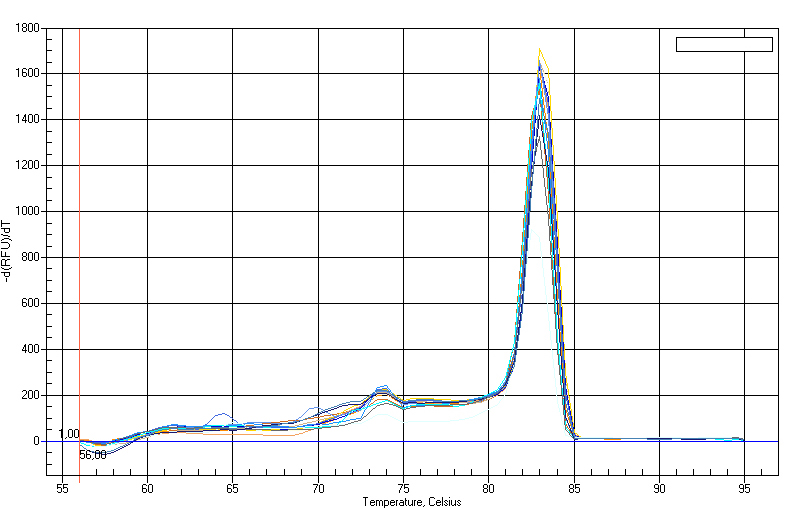

Supplement: Additional File 8 — Melting curve analyses obtained for the YWHAZ gene. Melting curve analyses image (jpg format) collected using the iQ5 Optical System Software 1.0 (Bio-Rad) during calibration experiments of the selected primer pair for the YWHAZ gene. Data were obtained using 1:5 dilutions of template cDNA (retrotranscribed from striped dolphin skin biopsy isolated total RNA) on a iQ5 machine (Bio-Rad). [file 1471-2199-7-32-S8.jpeg]

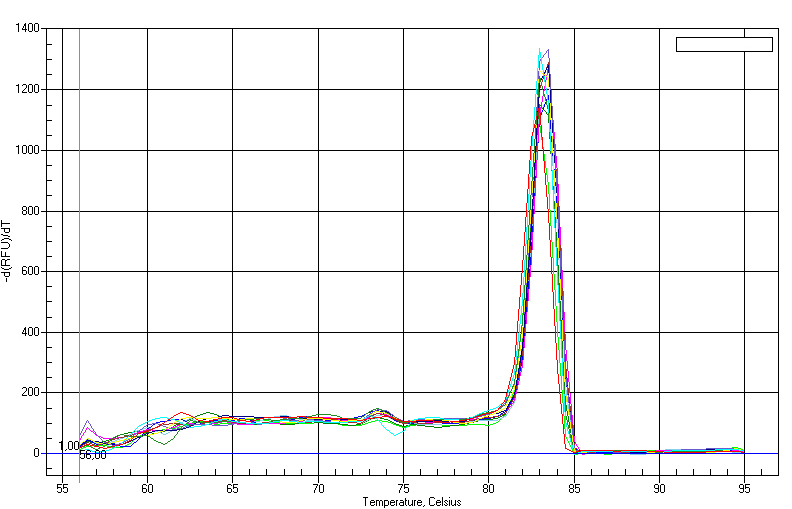

Supplement: Additional File 9 — Melting curve analyses obtained for the RPL4 gene. Melting curve analyses image (jpg format) collected using the iQ5 Optical System Software 1.0 (Bio-Rad) during calibration experiments of the selected primer pair for the RPL4 gene. Data were obtained using 1:5 dilutions of template cDNA (retrotranscribed from striped dolphin skin biopsy isolated total RNA) on a iQ5 machine (Bio-Rad). [file 1471-2199-7-32-S9.jpeg]

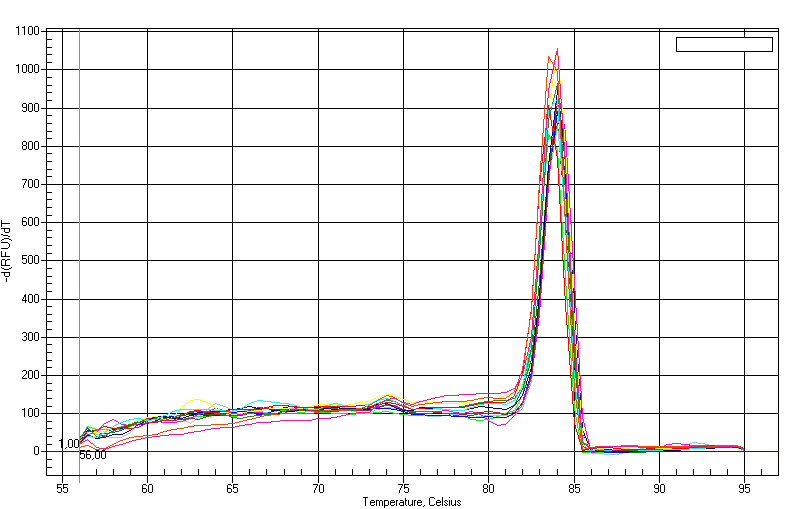

Supplement: Additional File 10 — Melting curve analyses obtained for the RPS18 gene. Melting curve analyses image (jpg format) collected using the iQ5 Optical System Software 1.0 (Bio-Rad) during calibration experiments of the selected primer pair for the RPS18 gene. Data were obtained using 1:5 dilutions of template cDNA (retrotranscribed from striped dolphin skin biopsy isolated total RNA) on a iQ5 machine (Bio-Rad). [file 1471-2199-7-32-S10.jpeg]
